# Inhibiting IGF1R-mediated Survival Signaling in Head and Neck Cancer with the Peptidomimetic SSTN_IGF1R_

**DOI:** 10.1158/2767-9764.CRC-22-0274

**Published:** 2023-01-19

**Authors:** Noah A. Stueven, DeannaLee M. Beauvais, Rong Hu, Randall J. Kimple, Alan C. Rapraeger

**Affiliations:** 1 Department of Human Oncology, University of Wisconsin–Madison, Madison, Wisconsin.; 2 Department of Pathology and Laboratory Medicine, School of Medicine and Public Health, University of Wisconsin–Madison, Madison, Wisconsin.

## Abstract

Previous studies have shown that the type I IGFR (IGF1R) suppresses apoptosis when it is autoactivated by coupling its extracellular domain to a matrix adhesion receptor complex consisting of syndecan-1 (Sdc1) and αvβ3 or αvβ5 integrin. We now report that head and neck squamous cell carcinoma (HNSCC) relies on this receptor complex. Disruption of the complex in HNSCC cells *in vitro* with a peptide mimetic of the organizer site in Sdc1 (called SSTN_IGF1R_) inactivates IGF1R, even in the presence of IGF1, and relieves the suppression of apoptosis signal-regulating kinase-1 (ASK1), dramatically reducing tumor cell survival. Normal epithelial cells do not assemble this receptor complex, require IGF1 to activate the IGF1R, and are refractory to SSTN_IGF1R_. *In vivo*, SSTN_IGF1R_ reduced the growth of patient-derived HNSCC tumors in immunodeficient mice by 85%–95%. IGF1R's assimilation into the matrix receptor complex, which is detected in these tumors using the proximity ligation assay (PLA), is quantitatively disrupted by SSTN_IGF1R_, coinciding with ASK1 activation. PLA also detects the IGF1R-containing receptor complex in the archival sections of tonsil carcinomas, whereas the adjacent benign epithelium is negative. Likewise, PLA screening of oropharyngeal and adenoid cystic tumor microarrays demonstrated that over 95% of the tumors contained this unique receptor complex with no detectable expression in benign tissue. These findings suggest that HNSCC upregulates and is highly dependent on IGF1R signaling via this adhesion receptor complex. Targeting this mechanism with novel therapeutics, including highly specific SSTN_IGF1R_, is likely to offer promising outcomes for patients with carcinoma.

**Significance:**

A newly developed biomarker reveals upregulation of an antiapoptotic IGF1R-integrin-syndecan receptor complex in head and neck cancer and documents disruption of the complex in patient-derived tumor xenografts (PDX) treated with the inhibitor SSTN_IGF1R_. A corresponding blockade in PDX growth in the presence of this inhibitor demonstrates that therapies designed to target this mechanism will likely offer promising outcomes for patients with head and neck cancer.

## Introduction

Head and neck squamous cell carcinoma (HNSCC) arises primarily in the mucosa of the oral cavity, pharynx, and oropharynx, with over 600,000 new cases diagnosed annually (1). Risk factors include smoking, alcohol consumption, and human papillomavirus infection (2). Patients with node-negative HNSCC are commonly treated with surgery or external beam radiation, whereas those with more advanced metastatic disease undergo combination therapy involving surgery, radiation, and cisplatin chemotherapy (2). A fraction of these patients may also benefit from cetuximab, a blocking antibody against the EGFR that is overexpressed in HNSCC, although the majority of patients are resistant to this therapy (3).

Another receptor tyrosine kinase (RTK) that has been linked to poor HNSCC disease outcomes is the type I IGFR (IGF1R; refs. 4–6). IGF1R and its ligand IGF1 have long been recognized as regulators of cell growth, survival, and transformation (7). In addition to HNSCC, IGF1R overexpression leads to poor prognosis in a number of other solid tumors, including colorectal, pancreatic, esophageal, ovarian, gastric, and non–small cell lung cancers (4–6, 8–14).

IGF1R protects tumor cells from stresses induced by cytotoxic cytokines, hypoxia, oxidative stress, and DNA damage (15), and downregulation of IGF1R sensitizes tumor cells to apoptosis (16). Suppression of apoptosis by the IGF1R has been linked to its association with the matrix receptor syndecan-1 (Sdc1; ref. 17). Syndecans organize RTKs and matrix-binding integrins into cell surface signaling complexes that are critical for cell invasion and survival, particularly in tumor cells (17–28). The kinases and integrins dock to specific binding motifs in the syndecan extracellular domains that can be mimicked by peptides (called “synstatins” or “SSTN”), leading to competitive blockade of receptor docking (17–21, 23, 26, 28). IGF1R docks to Sdc1 together with the αvβ3 or αvβ5 integrin (19). Peptide mimetics of the IGF1R and integrin docking site in Sdc1 (collectively called “synstatin-IGF1R” or “SSTN_IGF1R_”) inactivate IGF1R and the integrins in breast carcinoma, myeloma, and activated endothelial cells, disrupting cell growth and migration, activating apoptosis by reversing the suppression of apoptosis signal-regulating kinase-1 (ASK1) that occurs downstream of active IGF1R, and blocking tumor growth and tumor-induced angiogenesis in animal models (refs. 17–19; reviewed in ref. 28).

Here, we examined whether IGF1R is functionally linked to Sdc1 and αV-containing integrins in HNSCC. We used the proximity ligation assay (PLA; ref. 29) to detect the IGF1R-containing receptor complex in human HNSCC cell lines and in oropharyngeal and adenoid cystic tumor microarrays (TMA), and tested whether SSTN_IGF1R_ induced ASK1 activity and reduced cell growth and survival in HNSCC cell lines *in vitro* and patient-derived tumor xenografts (PDX) *in vivo*.

## Materials and Methods

### Reagents

**FIGURE 1:**
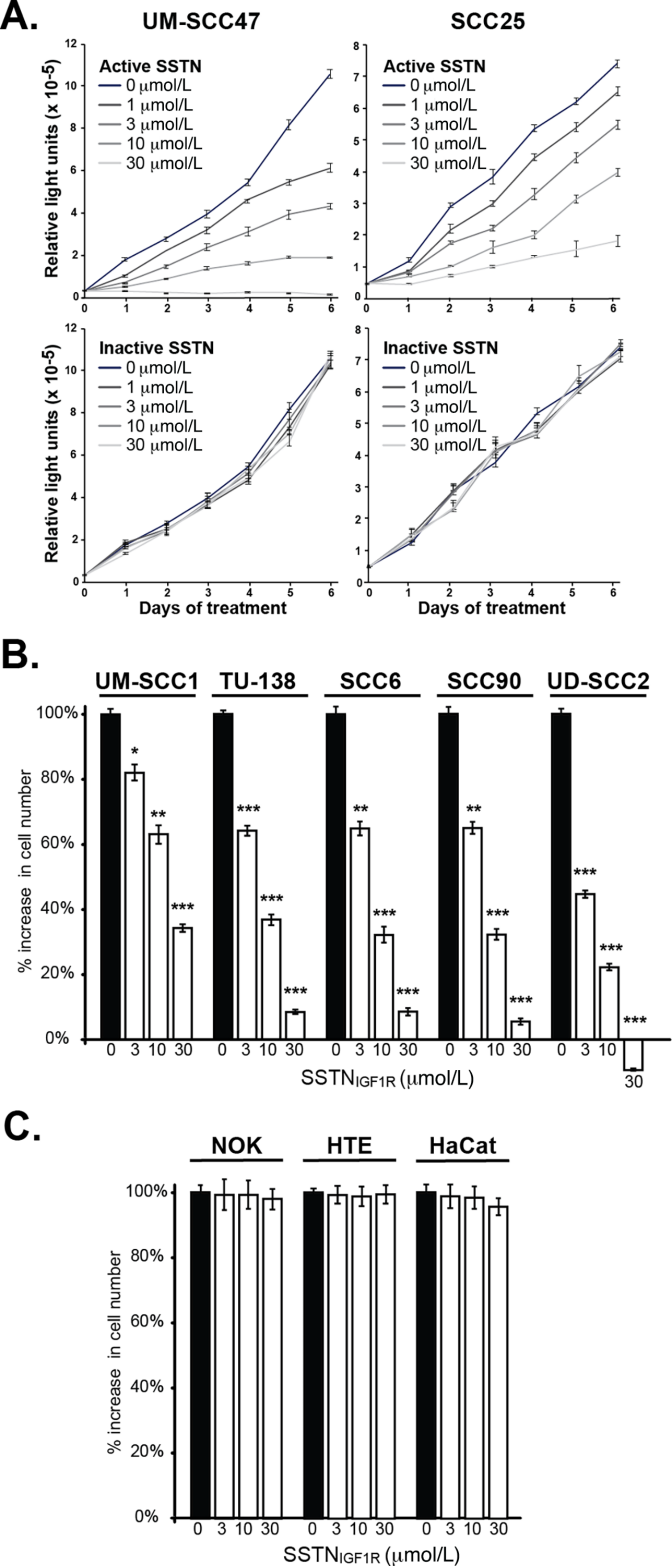
Quantification of HNSCC cell numbers following treatment with SSTN_IGF1R_. **A,** UM-SCC47 and SCC25 cell lines treated for up to 6 days with a concentration range of either active or inactive mSSTN_IGF1R_ (*n* = 1; quadruplicate wells/timepoint). **B,** Growth of five HNSCC cell lines quantified after 4 days treatment with up to 30 μmol/L active mSSTN_IGF1R_ (*n* = 4; ± SD, Student *t* test; *, *P* < 0.01; **, *P* < 0.001; ***, *P* < 0.0001). **C,** Growth of NOK, the, and HaCat nontransformed epithelial cells quantified after 4 days treatment with up to 30 μmol/L active mSSTN_IGF1R_ (*n* = 3; ± SD, Student *t* test, not significant). Cell numbers in **A,B,** and **C** are quantified using CellTiter-GLO and expressed as relative light units.

SSTN peptides (LifeTein LLC) were reconstituted in DMEM (Life Technologies) containing 20 mmol/L HEPES (Sigma-Aldrich) for *in vitro* studies or in phosphate-buffered 0.9% saline for use *in vivo*. Mouse SSTN_IGF1R_ (mSSTN_IGF1R_) and human SSTN_IGF1R_ (hSSTN_IGF1R_) peptides are equally effective in human cells (18, 19) and both peptides have been used in these studies. Active mSSTN_IGF1R_ (amino acids 92–119 of mouse Sdc1) and inactive mSSTN_IGF1R_ (amino acids 94–119) are used in Figs. 1 and 4B. Active hSSTN_IGF1R_ (amino acids 89–120 in human Sdc1) is used in all other experiments. Antibodies used were B-A38 (human Sdc1, Acris Antibodies) and a rabbit polyclonal described in ref. 30; LM609 (αvβ3 integrin), P1F6 (αvβ5 integrin), and NBP1-85746 (ITGAV) from Novus Biologicals; IGF1R-specific JBW902 (EMD Millipore), mAb 33255 (R&D Systems) and AF-305-NA from R&D Systems; JY202 (EMD Millipore), and Alexa647-conjugated mouse mAb K74-218 (BD Biosciences) against pY1131-IGF1R; 2E4 and D11C9 against ASK1, and 3765S (pThr845-ASK1) were from Thermo Fisher Scientific; 252355 (total JNK) was from R&D Systems and E.665.10 (pThr183/pTyr185 JNK) was from Thermo Fisher Scientific; D13E1 (p38MAPK), and 28B10 (pT183/Y185 p38MAPK) were from Cell Signaling Technology. The secondary antibodies used were Alexa488 donkey anti-rabbit IgG (Invitrogen), and Alexa488 donkey anti-goat IgG (Invitrogen). Isotype controls and anti-phosphotyrosine mAb PY20 were from BD Biosciences. DAPI was from Molecular Probes. CellTiter-GLO, ApoTox-GLO, and Caspase3/7-GLO were purchased from Promega. PathScan Stress and Apoptosis Signaling Antibody Array Kit was purchased from Cell Signaling Technology. Human recombinant DES(1–3)IGF1 was obtained from Gold Biotechnology, and the ASK1 inhibitor NQDI-1 (R&D Systems). The Duolink *in situ* PLA probe anti-goat PLUS, Duolink in Situ PLA Probe Anti-Rabbit Minus, and Duolink In Situ Detection Reagents Green were purchased from Millipore Sigma.

### Cell Culture

Parental tert-immortalized human normal oral keratinocytes (NOK) and human tonsillar epithelial cells (HTE) were provided by Paul Lambert (University of Wisconsin–Madison, Madison, WI). HaCaT keratinocytes (CVCL 0038) were provided by Dr. Peter LaCelle (Roberts Wesleyan College, NY, USA). Human SCC25 HNC (CVCL 1682) cells were obtained from the ATCC. UM-SCC47 (CVCL 7759), UM-SCC1 (CVCL 7707), TU-138 (CVCL 4910) and UM-SCC-6 (CVCL 7773), UPCI:SCC-090 (CVCL 1899), UD-SCC2 (CVCL E325), and UM-SCC22B (CVCL 7732) HNSCC cells were provided through the auspices of the Wisconsin Head and Neck cancer SPORE (https://hn-spore.wisc.edu). All cells, except for the NOKS and HTEs, were short tandem repeat profiled using Genetica LabCorp within 6 months of use. Cells were cultured at 37°C and 92.5% air/7.5% CO_2_. NOKs and HTEs were cultured in complete keratinocyte serum-free medium containing 100 U/mL penicillin and 100 μg/mL streptomycin (Life Technologies). Other cell lines were cultured as described previously (17–19, 23). Cells were passaged for a maximum of 3–4 months and screened for *Mycoplasma* approximately every 6 months by the Small Molecule Screening Facility at the University of Wisconsin Carbone Cancer Center using the R&D Systems Mycoprobe *Mycoplasma* Detection Kit (catalog no. CUL001B).

### Flow Cytometry

To measure cell surface receptor expression, suspended cells were incubated for 1 hour on ice with 1 μg of primary antibody per 5 × 10^5^ cells, washed, counterstained with Alexa488-conjugated goat secondary antibodies, and scanned on a Thermo Fisher Scientific Attune NxT benchtop cytometer. Cell scatter and propidium iodide (PI) staining profiles were used to gate live single-cell events.

### Immunoprecipitations

Immunoprecipitation of IGF1R with or without competing SSTN peptide was carried out as described previously (18, 19) using 0.5–1.0 mg of input/sample and lysis buffer supplemented with HALT protease and phosphatase inhibitor cocktail (Thermo Fisher Scientific).

### Cell Proliferation Assays

Cells (1–2 × 10^3^/well) were plated in 96-well plates in complete culture medium in the presence or absence of the SSTN_IGF1R_ peptide for 72–120 hours, or alternatively in the presence or absence of the SSTN_IGF1R_ peptide or ASK inhibitor for 24 hours. Cell growth was measured against a standard curve using CellTiter-GLO, in accordance with the manufacturer's instructions.

### Caspase Assay and Apoptosis Marker Array

The activation of stress/apoptosis markers was analyzed as described previously (17). Caspase activity was measured in cells in complete culture medium with SSTN_IGF1R_ or ASK inhibitor for 24 hours using Caspase 3/7-GLO in accordance with the manufacturer's instructions.

### Tumor Formation in Animals

Animal studies were approved by the University of Wisconsin–Madison Institutional Animal Care and Use Committee in accordance with the NIH guidelines. UW-SCC22 or UW-SCC64 PDXs derived from head and neck tumors (31, 32) were provided by Wisconsin Head and Neck Cancer SPORE (https://hn-spore.wisc.edu). Minced tissue isolated from host mice was subcutaneously injected into both flanks of 6–8 weeks old female, athymic *Foxn1^nu^* outbred nude mice (Harlan Laboratories). After 1 week, animals were randomized and surgically implanted with Alzet (Durect Corp.) osmotic pumps (Model 2004) delivering 0.25 μL/hour of either PBS or PBS containing 4 mmol/L hSSTN_IGF1R_ for 4 weeks. Animals were humanely sacrificed, bled, and tumors were removed and fixed in 4% paraformaldehyde before paraffin embedding and sectioning.

### Serum Peptide and IGF1 Analysis

SSTN_IGF1R_ levels in the serum of treated animals were measured in triplicate using an AB Sciex QTrap 5500 mass spectrometry system at the UW School of Pharmacy and quantified by comparison with a standard curve of peptide in mouse serum. IGF1 levels in the serum of animals bearing UW-SCC22 or UW-SCC64 tumors were measured using an ELISA kit from Thermo Fisher Scientific (EMIGF1) and compared with a mouse IGF1 standard according to the manufacturer's instructions.

### Immunofluorescent Receptor Staining and PLA

#### Cultured Cells

UM-SCC47 cells treated with or without the SSTN_IGF1R_ peptide for 6 hours were fixed in 4% paraformaldehyde, permeabilized in 0.5% Triton X-100 in 1× CMF-PBS (pH 7.4), and blocked for 1 hour at room temperature in a 3% BSA/CMF-PBS solution. Cells were stained with 1 μg/mL anti-Sdc1 polyclonal antibody, 2 μg/mL ITGAV antibody (NBP1-85746), or 4 μg/mL antibody to IGF1R (AF-305-NA) for 1 hour at room temperature, followed by incubation with 4 μg/mL Alexa488-conjugated secondary antibodies for 1 hour at room temperature. The nuclei were stained with 3 μmol/L DAPI (Molecular Probes) in 1× CMF-PBS (pH 7.4) for 10 minutes. For PLA, antibodies against ITGAV and IGF1R were used as described previously, followed by staining with PLA reagents according to the manufacturer's instructions.

#### Tumor Sections

Paraffin-embedded PDX sections, sections of deidentified archival tumors from patients with tonsil carcinoma, or TMAs assembled from patients with deidentified oropharyngeal (TMA-01, TMA-02) or adenoid cystic (TMA-03) carcinoma were deparaffinized and subjected to antigen retrieval by heating in 10 mmol/L sodium citrate (pH 6.0) with 0.05% Tween 20 until boiling and then maintained at 99°C for 20 minutes before cooling to room temperature. The sections were then used for receptor and PLA staining, as described above. Fluorescent images were acquired using either a Zeiss PlanAPOCHROMAT 20X objective (0.8 NA) or 40X (1.4 NA) and a Zeiss AxioCam Mrm CCD camera on a Zeiss Axio Imager.M2 microscopy system. CellProfiler 3.1.5 (Carpenter Lab, Broad Institute of Harvard and MIT) was used to quantify nuclei and PLA signals with threshold limits based on relevant comparative controls. Cell Profiler software analysis was checked by direct comparison with visual quantification of PLA dots or DAPI-stained nuclei in test images to accurately quantify PLA dots per cell and to exclude false or background fluorescence. The specificity of the antibodies used and the concentrations of PLA reagents used were optimized on test samples before use on experimental sections.

### Data Availability

The data generated in this study are available for this article.

## Results

The reliance of HNSCC on IGF1R coupled to Sdc1 was initially examined by screening the UM-SCC47 and SCC25 tongue carcinoma cell lines for their response to active or inactive mSSTN_IGF1R_ peptides. These peptides were extensively defined previously and shown to disrupt this mechanism in either mouse or human tumor and/or vascular endothelial cells (18, 19). This screen shows that both cell lines appear to rely on this receptor complex; both exhibited reduced cell numbers in the presence of the active peptide administered over 6 days, with an IC_50_ in the range of 1–10 μmol/L, whereas the inactive peptide had no effect (Fig. 1A). This screen was extended to other cells using a 4-day timepoint for a comparative analysis with normal epithelial cells. UM-SCC1, TU-138, SCC6, SCC90, and UD-SCC2 HNSCC cell lines exhibited reduced growth in the presence of active mSSTN_IGF1R_ (Fig. 1B). In contrast, NOKs, normal HTE cells, and human epidermal HaCaT keratinocytes (Fig. 1C) were unaffected by the peptide.

**FIGURE 2:**
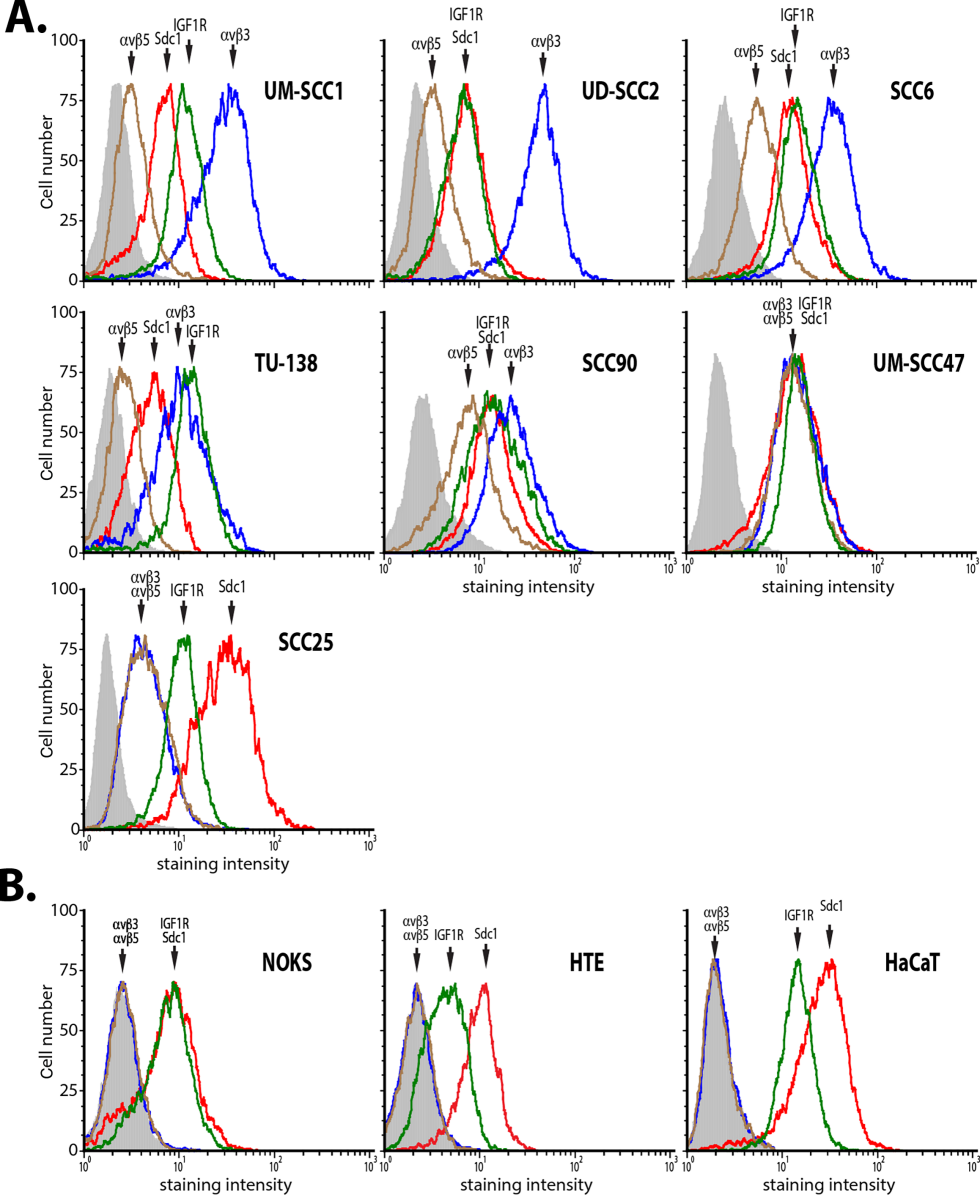
Cell Surface expression of Sdc1, IGF1R, and αvβ3 or αvβ5 integrins in HNSCC and nontransformed epithelial cells. Flow cytometry analysis of HNSCC (**A**) and nontransformed epithelial cells (**B**). Data shown are a representative example of duplicate scans of each cell line.

Docking of IGF1R with Sdc1 requires the coassembly of Sdc1 with the αvβ3 or αvβ5 integrin (19). This predicts that the peptide-responsive HNSCC cells coexpress all three of these receptors. Accordingly, flow cytometry analysis demonstrated that the HNSCC cells analyzed in Fig. 1 expressed Sdc1 and αvβ3 or αvβ5 integrins along with IGF1R (Fig. 2A). In contrast, whereas Sdc1 and IGF1R are expressed on the peptide-resistant NOKs, HTEs, and HaCaT keratinocytes, these cells lack surface expression of the integrins (Fig. 2B).

**FIGURE 3:**
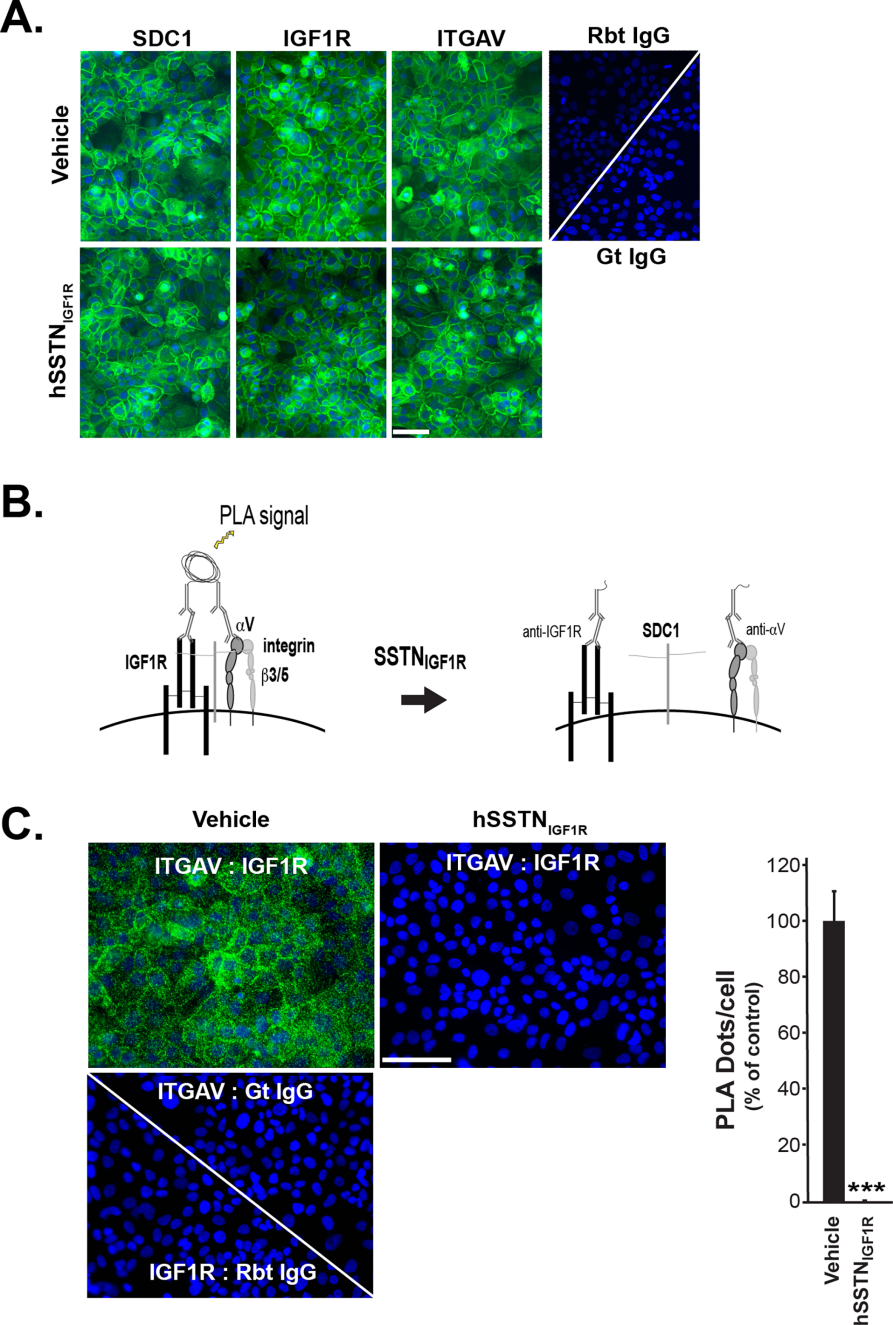
Detection of assembled receptor complex using proximity ligation assay. **A,** Staining of fixed UM-SCC47 cells pretreated for 6 hours with vehicle or 30 μmol/L hSSTN_IGF1R_, then stained for SDC1, IGF1R, or ITGAV compared with control rabbit (Rbt) or goat (Gt) IgG (bar = 50 μm). Data are representative of duplicate experiments. **B,** Model of PLA between ITGAV and IGF1R. **C,** PLA between ITGAV and IGF1R (ITGAV:IGF1R) in fixed UM-SCC47 cells pretreated for 6 hours with vehicle (left top) or 30 μmol/L hSSTN_IGF1R_ (right top), compared with PLA between ITGAV and control goat IgG or IGF1R and control rabbit IgG in vehicle-treated cells (bottom left; bar = 50 μm). Individual cells are identified by DAPI-stained nuclei. PLA is quantified as dots per cell across 15 images over three experiments and graphed as a percentage of PLA observed in untreated cells for ease of comparison (± SD, Student *t* test; ***, *P* ≤ 0.001). For reference, the average for the untreated cells was 72 dots per cell.

As a third step in this screen, we verified abundant cell surface expression of these three receptors on UM-SCC47 cells by indirect immunofluorescence staining (Fig. 3A), then used these cells to refine the proximity ligation assay to screen for assembly of the three receptors into a single complex and the disruption of this complex by SSTN_IGF1R_ (ref. 29; see model in Fig. 3B). Importantly, no change in cell surface expression of any receptor is observed in cells treated with SSTN_IGF1R_ compared with vehicle alone (Fig. 3A). Next, to determine whether IGF1R and integrin are indeed paired via their coassembly with syndecan, we detected their close apposition using PLA. Note that the integrins are observed in the PLA by staining their αV subunit (ITGAV) because our ultimate goal is to develop PLA for use on paraffin-embedded tumor sections and commercial antibodies to the integrin β3 or β5 subunits that work on paraffin-embedded specimens could not be identified. PLA staining detected significant colocalization of IGF1R and ITGAV in untreated UM-SCC47 cells, but the signal is lost in cells pretreated for 6 hours with hSSTN_IGF1R_ (Fig. 3C) despite the abundant expression of all three receptors at the cell surface (cf. Fig. 3A).

**FIGURE 4:**
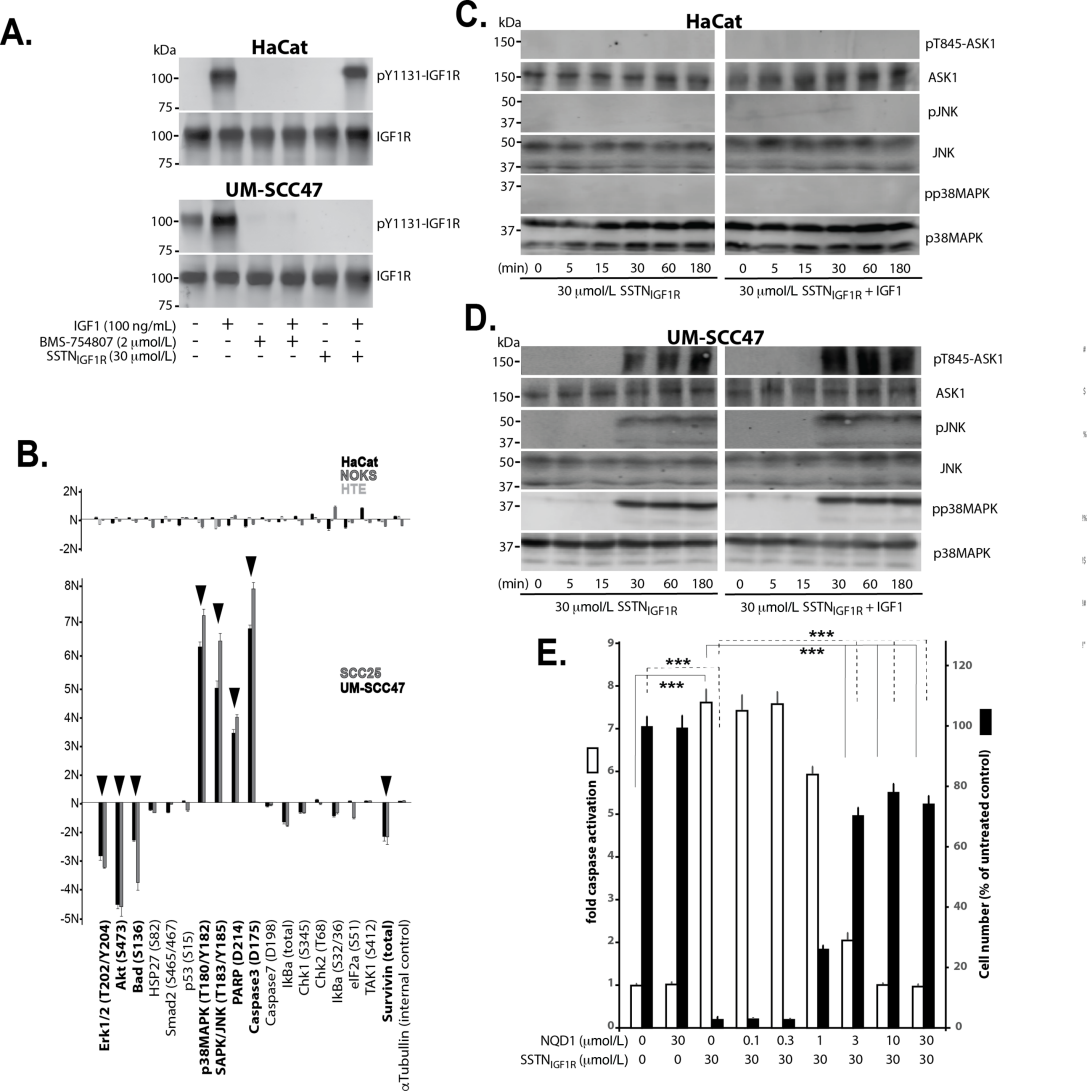
Analysis of ASK-mediated apoptosis activated by SSTN_IGF1R_ in HNSCC cells. **A,** Active IGF1R is detected by staining with anti-p1131 on Western blots following immunoprecipitation from HaCat or UM-SCC47 cells after 3 hours treatment with or without 100 ng/mL DES(1–3)IGF1 in the presence of IGF1R inhibitor (2 μmol/L BMS-754807) or 30 μmol/L hSSTN_IGF1R_. Data are representative of three experiments. **B,** Cell lysates from UM-SCC47 and SCC25 HNSCC cells grown for 16 hours in the presence or absence of 30 μmol/L mSSTN_IGF1R_ are compared with nontransformed HaCat, NOK, or HTE cells using an antibody array to analyze the relative fold changes in expression and/or phosphorylation levels of stress and apoptotic markers compared with normal (N) levels observed in vehicle-treated cells. Data represent duplicate experiments and duplicate samples/experiment; significant increases or decreases are noted (boldface, arrowheads; ± SD, Student *t* test). Activation of p38MAPK (pp38MAPK), JNK (pJNK), and ASK1 (pT845ASK1) are analyzed in UM-SCC47 cells treated for up to 3 hours with 30 μmol/L hSSTN_IGF1R_ in the absence (**C**) or presence (**D**) of DES(1–3)IGF1. Data shown are representative of triplicate experiments. **E,** Cell numbers compared with relative caspase activity is quantified in UM-SCC47 cells treated for 24 hours with vehicle or 30 μmol/L hSSTN_IGF1R_ in the presence of increasing concentrations of ASK inhibitor NQD1. Cell numbers are determined using the CellTitre Glo assay, expressed as relative light units and graphed as a percentage of cells treated with vehicle alone. Relative caspase activity is measured using the Caspase 3/7-GLO kit and expressed as fold change from cells treated with vehicle alone. Data represent triplicate samples from triplicate experiments (± SD, Student *t* test; ***, *P* ≤ 0.001).

Previous studies have shown that IGF1R is constitutively activated in an IGF1-independent manner when engaged with Sdc1 and this is blocked by SSTN_IGF1R_ (17, 19). More importantly, this block by the peptide extends to IGF1R activated by IGF1 as well, although the mechanism for inhibiting ligand-mediated activation is not understood (17). Extending this finding to HNSCC, constitutively active IGF1R is identified in UW-SCC47 cells by staining for pY1131 in the absence of IGF1, whereas IGF1R was not active in HaCat cells used as a nontransformed control (Fig. 4A). Although this is likely to be constitutive activation due to IGF1R incorporation into the adhesion receptor complex with Sdc1, we cannot rule out the possibility that activation is due to autocrine IGF1 production by the UM-SCC47 cells. As expected, Y1131 phosphorylation is stimulated by exogenous DES(1–3)IGF1 in both cell lines and inhibited by the IGF1R kinase inhibitor BMS-754807 (Fig. 4A). DES(1–3)IGF1 is a truncated IGF1 variant with greatly reduced affinity for IGF1-binding proteins (IGFBP) and was used to avoid potential effects of IGFBPs secreted by the tumors cells. IGF1-mediated activation of IGF1R in HaCat cells is unaffected by SSTN_IGF1R_, indicating that IGF1R is activated in its classic ligand-dependent manner and acts independently of Sdc1 in these cells. However, IGF1-mediated activation of IGF1R is blocked by SSTN_IGF1R_ in the tumor cells (Fig. 4A), indicating that activation of IGF1R with or without IGF1 in the tumor cells is highly dependent on its linkage to Sdc1.

Prior work also demonstrated that IGF1R activated by its linkage to Sdc1 suppresses apoptosis by inhibiting the activation of ASK1 (17, 19). ASK1 is present in the Sdc1/IGF1R/integrin complex where phosphorylation by IGF1R, in part, inhibits its autoactivation (17, 33). This suppression is reversed when tumor cells are treated with SSTN_IGF1R_, with the expression of apoptotic markers following within an hour and increasing over the ensuing 24 hours (17). Screening of an apoptotic antibody array using lysates of UM-SCC47 or SCC25 HNSCC cells treated for 16 hours with 30 μmol/L SSTN_IGF1R_ revealed a significant change in apoptotic markers when the tumor cells were treated with the peptide, as evidenced by significant increases in activated caspase 3 and PARP, and reduced levels of survivin and phosphorylated Erk, Akt, and Bad (Fig. 4B). The NOK, HTE, and HaCaT keratinocyte controls showed little or no response to the peptide (Fig. 4B).

Inhibition of IGF1R signaling by SSTN_IGF1R_ has been shown to activate the p38MAPK and JNK signaling cascades downstream of ASK1 (17), enzymes known to activate apoptosis (34). Similarly, the inhibition of IGF1R in the HNSCC tumor cells is accompanied by major increases in active p38MAPK and JNK (Fig. 4B–D). Examining the temporal activation of this MAPK cascade, we observed that active ASK1 (pT845), pp38MAPK, and pJNK increased 30 minutes after the treatment of UM-SCC47 cells with SSTN_IGF1R_ (Fig. 4C). This increase is not suppressed by exogenous IGF1 (Fig. 4D), consistent with the inability of IGF1 to activate the IGF1R in the presence of SSTN_IGF1R_. To confirm that induction of apoptosis via ASK activation explains the effects of SSTN_IGF1R_ on the growth of tumor cells, UM-SCC47 cells were grown in the presence of SSTN_IGF1R_ and the ASK inhibitor NQD1. Increasing concentrations of NQD1 up to 30 μmol/L gradually reduce caspase 3 activation induced by 30 μmol/L SSTN_IGF1R_, which was mirrored by 80% restoration of cell growth in the SSTN-treated cells (Fig. 4E).

**FIGURE 5:**
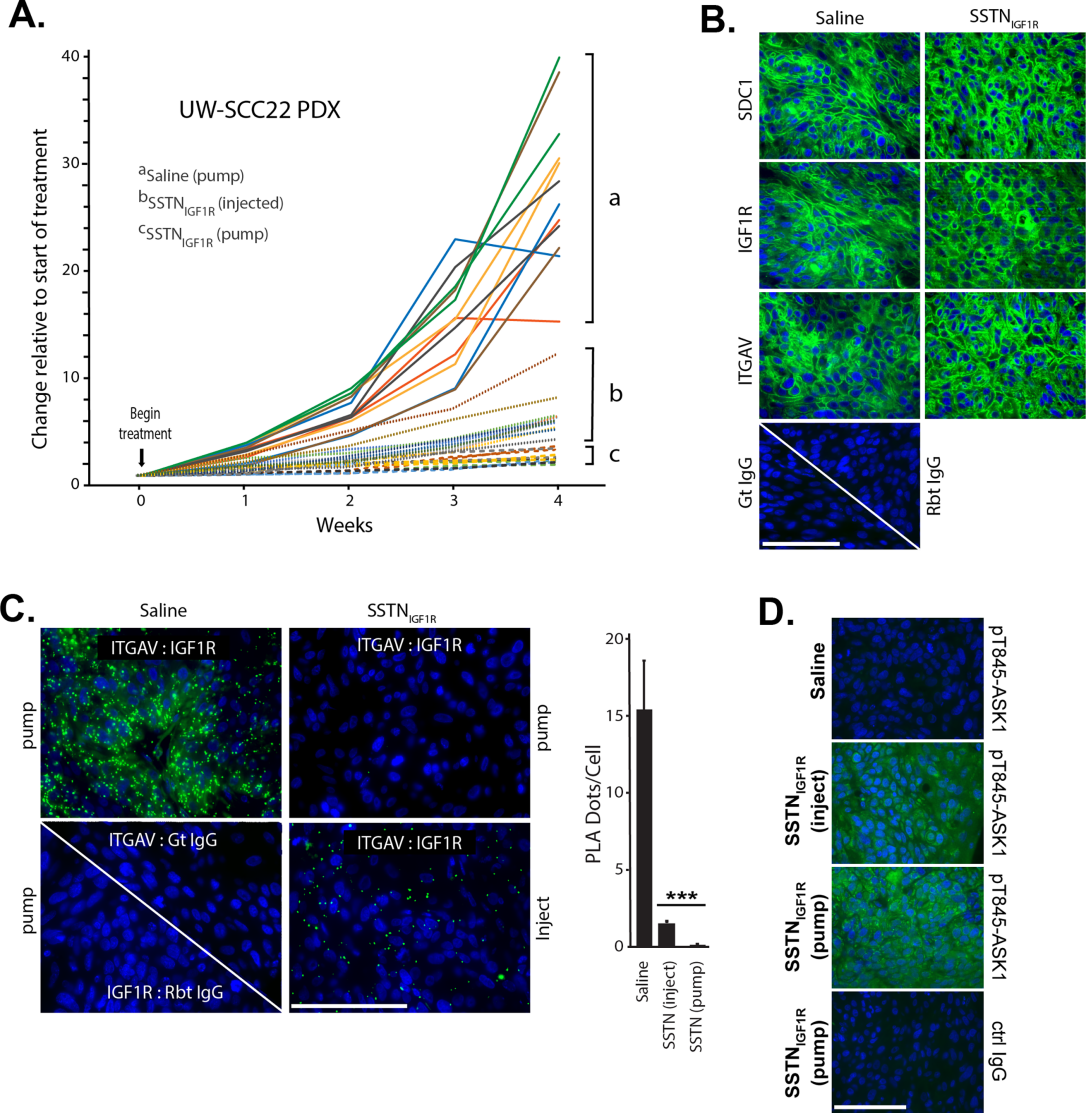
Response of PDX UW-SCC22 to hSSTN_IGF1R_. **A,** UW-SCC22 PDXs implanted subcutaneously as bilateral tumors on the flanks of nude mice were allowed to establish for 1 week, followed by systemic delivery of 4.3 mg/kg/day hSSTN_IGF1R_ via Alzet pump, saline alone delivered by Alzet pumps, or twice weekly subcutaneous injection of hSSTN_IGF1R_ in saline. Tumor size (0.524 × length × width^2^) was documented for 4 weeks. **B,** Fixed, paraffin-embedded sections of tumors treated for 4 weeks with either saline or hSSTN_IGF1R_ (pump delivery) are stained for expression of SDC1, IGF1R, or ITGAV compared with control goat (Gt) or rabbit (Rbt) IgG (bar = 50 μm). **C,** PLA is conducted between IGF1R and ITGAV in the tumors treated for 4 weeks either with saline, hSSTN_IGF1R_ in Alzet pumps (pump) or tumors subjected to biweekly hSSTN_IGF1R_ injection (inject) and is graphed as dots per cell (visualized using DAPI-stained nuclei). As controls, PLA is conducted using goat anti-IGF1R and nonspecific rabbit IgG or rabbit anti-ITGAV and nonspecific goat IgG (bar = 50 μm; ± SD, Student *t* test; ***, *P* ≤ 0.001). **D,** Sections of tumors treated with saline or hSSTN_IGF1R_ delivered by injection or pump for 4 weeks are stained with anti-pT845-ASK1 antibody compared with nonspecific IgG control (Ctrl; bar = 50 μm).

SSTN_IGF1R_ has a half-life (∼24 hours) which allows its use as a therapeutic agent *in vivo* (17). Accordingly, the peptide was delivered at 4.3 mg/kg/day to treat the UW-SCC22 HNSCC PDX for a period of 4 weeks, either by constant infusion via an Alzet pump or twice weekly subcutaneous injection. Tumors in control animals treated with saline grew from 102 ± 21 mm^3^ to 2,767 ± 631 mm^3^ over this period, whereas tumors treated with pump-delivered SSTN_IGF1R_ grew to 270 ± 49 mm^3^, a reduction of approximately 94% (*P* < 0.001; Fig. 5A). Tumors treated with the injected peptide grew to 664 ± 138 mm^3^, a reduction of 79% (*P* < 0.001) compared with controls. Analysis of peptides in the plasma of treated animals following sacrifice revealed a higher steady-state concentration when delivered by pump (32 μmol/L) compared with injection (13 μmol/L), explaining the disparity in tumor growth. Staining of fixed, paraffin-embedded tumor sections demonstrated abundant expression of Sdc1, IGF1R, and αV-containing integrin in both the saline-treated and SSTN_IGF1R_ (pump)-treated tumors (Fig. 5B; the tumors selected for staining are marked with asterisks in Fig. 5A). However, whereas PLA detected abundant ITGAV and IGF1R pairing in the saline-treated tumors, pairing was reduced by 90% in the tumors in which the plasma SSTN_IGF1R_ concentration was 13 μmol/L and reduced to nearly undetectable levels when it reached 32 μmol/L due to pump-mediated delivery (Fig. 5C). Positive staining for pT845-ASK-1 confirmed its activation in tumors treated with SSTN_IGF1R_ (Fig. 5D).

**FIGURE 6:**
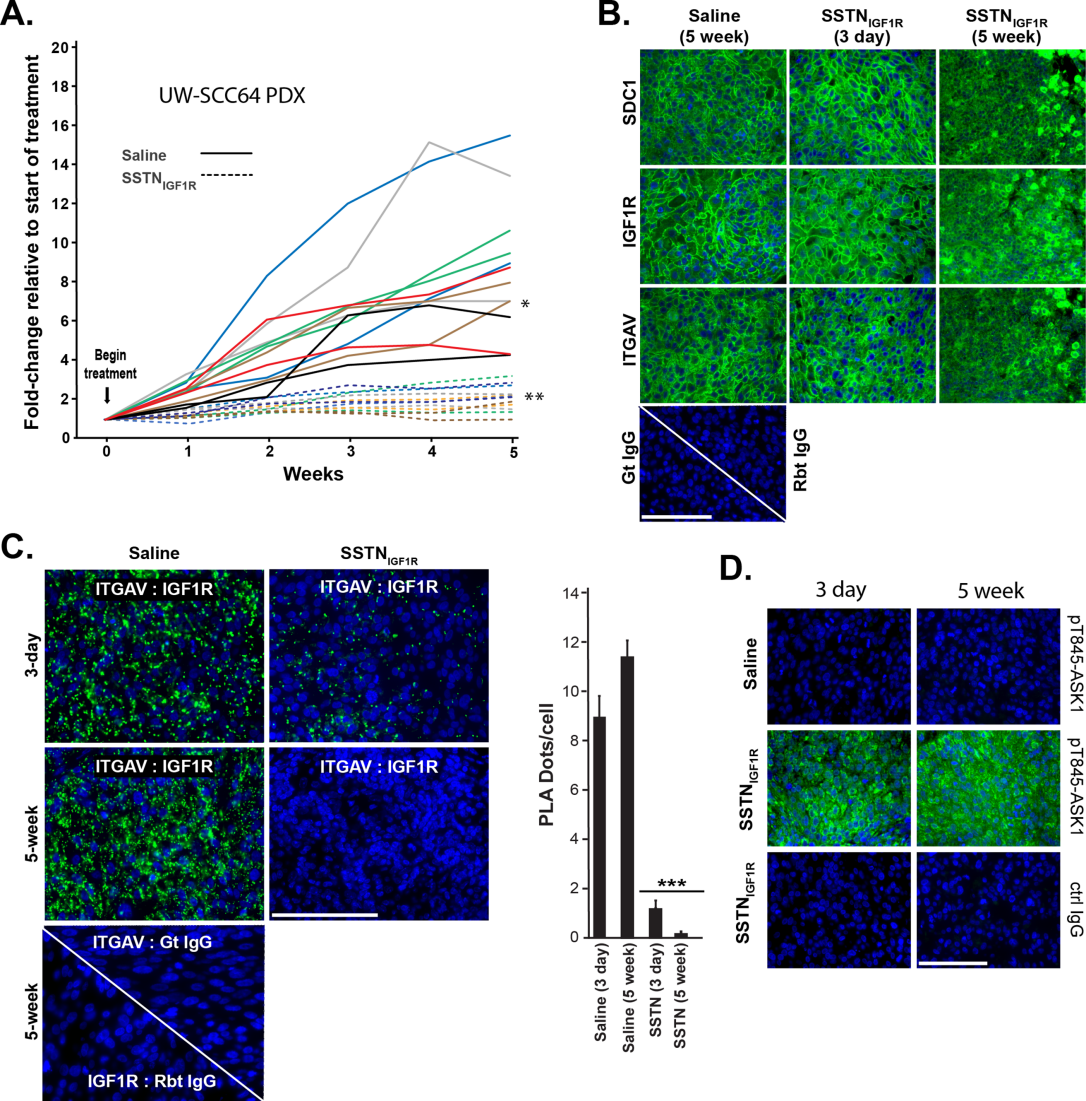
PLA and ASK1 activation in PDX UW-SCC64. Bilateral UW-SCC64 PDXs were implanted and treated with either saline or hSSTN_IGF1R_ delivered by Alzet pump (**A**), fixed, embedded, and sections stained for receptor expression (**B**) or for PLA (**C**) as described for UW-SCC22 PDXs in figure legend 5; a cohort of mice were sacrificed after 3 days of treatment to analyze the effects of hSSTN_IGF1R_ after this short exposure (bar = 50 μm; ± SD, Student *t* test; ***, *P* ≤ 0.001). **D,** Sections of tumors treated with hSSTN_IGF1R_ or saline for 3 days and 5 weeks are stained with anti-pT845-ASK1 antibody compared with nonspecific IgG control (Ctrl; bar = 50 μm).

This experiment was repeated using a second PDX (UW-SCC64), with the peptide delivered by the pump only. SSTN_IGF1R_ reduced tumor growth by 85% (*P* < 0.001) compared with saline-treated controls (Fig. 6A). Abundant expression of Sdc1, IGF1R, and ITGAV was observed in control tumors and tumors harvested after 3-day SSTN treatment, but was reduced in tumors treated for 5 weeks with SSTN_IGF1R_ likely due to necrosis (Fig. 6B). PLA analysis of control tumors indicated extensive pairing of ITGAV and IGF1R, which was already reduced by 80% after 3-day treatment with SSTN and essentially abolished in the 5-week tumors (Fig. 6C), accompanied by pT845 staining indicative of ASK-1 activation as early as the 3-day timepoint (Fig. 6D).

Current therapeutic approaches targeting the IGF1R, such as blocking antibodies and kinase inhibitors, do not distinguish between normal and transformed cells, thereby causing increases in plasma IGF1 by inhibiting the IGF1 feedback loop in the hypothalamus, which depends on IGF1R (35). In contrast, serum levels of IGF1 in animals subjected to SSTN_IGF1R_ while bearing UW-SCC22 or UW-SCC64 tumors showed no change, supporting the notion that the peptide affects IGF1R in tumors but not in normal physiologic processes (Table 1).

**TABLE 1:** Serum IGF1 levels in treated mice

	IGF1 (ng/mL) ± SD
**UW-SCC22**	
Saline (control)	34.8 ± 1.0
SSTN_IGF1R_	34.6 ± 0.9
**UW-SCC64**	
Saline (control)	34.0 ± 0.9
SSTN_IGF1R_	33.7 ± 1.1

NOTE: Blood samples (*n* = 6) are from animals treated with hSSTN_IGF1R_ or saline delivered by Alzet pumps.

**FIGURE 7:**
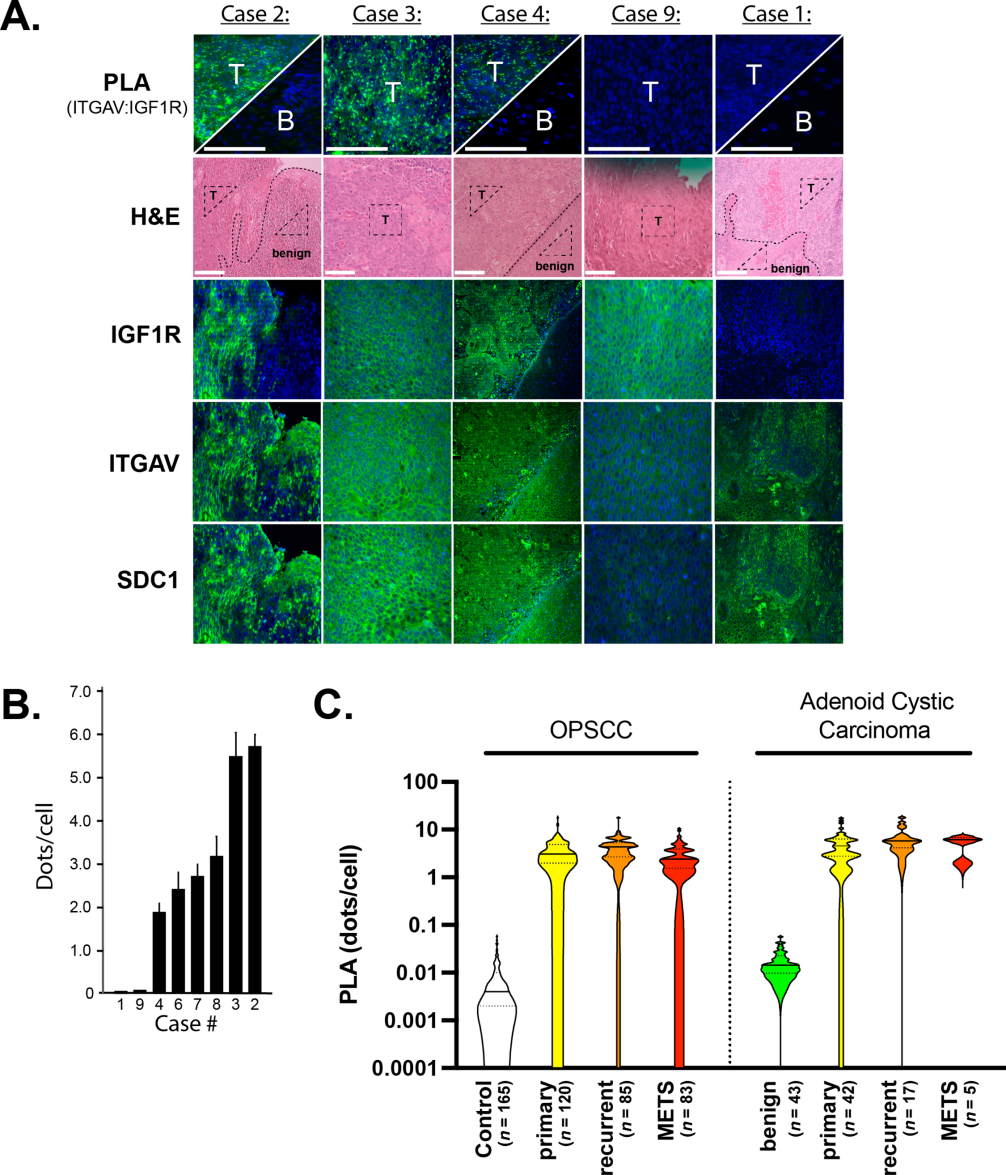
PLA screen of archival human head and neck carcinomas. **A,** Consecutive serial paraffin sections of eight archival human tongue tumors were stained for Sdc1, ITGAV, or IGF1R, or subjected to PLA to detect coupling of ITGAV and IGF1R. Shown are three of the six positive PLA cases (cases #2–4) representative of the range of observed staining, including the two tumor cases containing adjacent benign tissue, along with the two cases (cases #9 and #1) demonstrating negative PLA. Tissue histology as viewed by hematoxylin and eosin (H&E) staining and benign tissue and the tumor margins are marked (dashed line), if present. Triangles within the benign tissue (B) or tumor (T) identify the areas examined by PLA at higher magnification (bar = 50 μm). **B,** Quantification of ITGAV:IGF1R PLA intensity (PLA dots/cell) in the eight cases of tongue carcinoma arranged in order of intensity (PLA is quantified as dots per cell across 15 images over three experiments). Case #5 was excluded because of high endogenous fluorescence. **C,** Quantification of ITGAV:IGF1R PLA conducted on three tissue microarrays [one adenoid cystic carcinoma microarray and two oropharyngeal squamous cell carcinoma (OPSCC) microarrays]. PLA quantified as fluorescent dots per cell for each tissue based on the averaged staining of three microarray sections and expressed as a violin plot (solid line is the median and dotted lines are quartiles). Range for the tumors is 0.000–17.704 dots per cell and benign tissue is 0.004–0.056. Negative control staining of the same sections ranges from 0.000 to 0.057 dots per cell.

Given the efficacy of SSTN_IGF1R_ against HNSCC cell lines and PDXs, we used PLA as a biomarker for the Sdc1:IGF1R:ITGAV receptor complex in archival tumors. Nine cases of tonsillar carcinoma were initially analyzed using PLA, although case #5 was ultimately excluded because of high endogenous fluorescence that precluded further study. Cases #1 and #9, representing two tumors that exhibited negative PLA, and cases #2–4 representing the spectrum of positive PLA results observed in the remaining six cases are shown in Fig. 7A. Relative levels of ITGAV:IGF1R pairing observed in each of the eight cases are shown in Fig. 7B. All three receptors (Sdc1, ITGAV, and IGF1R) showed strong membranous and/or cytoplasmic staining in the three PLA-positive tumors (Fig. 7A). However, one or more of the three required receptors showed low to negative expression in the two PLA-negative tumors, with the IGF1R in case #1 and both Sdc1 and ITGAV in case #9 expressed below our detection levels (Fig. 7A). PLA also failed to detect the receptor complex in benign epithelial tissue adjacent to PLA-positive tumors, represented by cases #2 and #4; moderate expression of Sdc1 and ITGAV is observed in the benign tissue, but levels of IGF1R were below our detection limit (Fig. 7A).

Finally, we used the PLA technique to screen the proximity of ITGAV and IGF1R in archival oropharyngeal and adenoid cystic TMAs containing triplicate cores of tumor and matched normal tissues, when available. Samples were categorized as primary, recurrent, or metastatic (Supplementary Table S1). Oropharyngeal and adenoid cystic carcinomas showed similar receptor complex expression (Fig. 7C). Combined, greater than 95% of the primary tumor samples (*n* = 164), recurrent tumors (*n* = 104), and metastases to lymph nodes or other sites (*n* = 85) exhibited positive PLA, defined as > 0.2 PLA dots per cell (range, 0.2–17.9). Staining of benign salivary gland tissue from patients with adenoid cystic carcinoma (*n* = 43) or control staining of oropharyngeal tumors was uniformly negative (range, 0.0–0.06 PLA dots/cell; Fig. 7C).

## Discussion

This work reveals the prevalent expression in HNSCC of a previously described three-receptor complex consisting of IGF1R, the αV-containing αvβ3 or αvβ5 integrin and Sdc1 that suppresses apoptosis in cancer cells (refs. 17–19; reviewed extensively in ref. 28). SSTN_IGF1R_ mimics the defined site in the extracellular domain of Sdc1 responsible for the interaction between IGF1R and integrins (18, 19). It competes for this docking, disrupts the complex, and reverses the suppression of apoptosis, which depends on the active IGF1R (18, 19).

IGF1R is incorporated into the matrix adhesion receptor complex only if the αvβ3 or αvβ5 integrin is engaged with Sdc1 (19). Therefore, a positive PLA signal between the IGF1R and the alpha-V subunit of integrin indicates that the full functional complex is assembled. This signal shows that over 95% of archival human oropharyngeal and adenoid cystic carcinoma tumors express this complex, including primary, recurrent, and metastatic tumors. This is especially striking given the complete lack of the receptor complex in adjacent benign tissues, mirroring its absence in normal epithelial cells in culture. It seems reasonable to speculate that the receptor complex does have a role in normal epithelia at some point, perhaps during development or in wound healing, but its lack of expression in epithelia from mature organs clearly reduces the potential for off-target effects by SSTN_IGF1R_ and supports a high degree of specificity of the peptide toward carcinomas. The reasons for its absence in normal epithelia seem to vary; benign tonsillar epithelial cells *in vivo* appear to express Sdc1 and the alpha-V integrins, but lack IGF1R, whereas normal epithelial cells in culture express Sdc1 and IGF1R, but lack αvβ3 and αvβ5 integrins, potentially reflecting divergence that occurs during growth selection *in vitro*. However, whether formation of the complex depends solely on regulation of receptor expression or also on factors that control receptor trafficking to the cell surface, influence their incorporation into specialized lipid domains in the plasma membrane, or control their assembly with intracellular signaling components is a possibility that remains to be studied.

The initial description of this receptor complex has been one of the numerous studies over the past several decades to demonstrate that signaling by RTKs is closely coordinated with cell-matrix adhesion. Syndecans have been shown to be the organizers of integrin-RTK complexes involving IGF1R, VEGFR2 (KDR), HER2, EGFR, and RON kinase (reviewed in ref. 28) with others likely yet to be discovered. RTK activation in these mechanisms depends on engagement with syndecan and is typically ligand independent, setting these mechanisms apart from classical mechanisms of RTK activation. As confirmed here, assembly of IGF1R with Sdc1 and integrins in tumor cells, together with the subsequent clustering that occurs when Sdc1 engages the matrix, constitutively activates IGF1R kinase, even in the absence of IGF1 (19). Accordingly, antibodies that block IGF1 binding do not block this mechanism (17), allowing it to escape therapeutics designed to disrupt ligand activation of the IGF1R. This indicates a major difference between transformed and benign epithelial cells: IGF1R activation in cultured normal epithelial cells depends on IGF1 and is not affected by SSTN_IGF1R_, confirming the specificity of this peptide competitor for IGF1R assembled with Sdc1. In contrast, IGF1R activation in tumor cells is strictly dependent on Sdc1, even if IGF1 is present, and is therefore blocked by SSTN_IGF1R_. This suggests that IGF1R is assembled into an activation-signaling architecture in tumor cells that is significantly different from that in normal cells and that IGF1R activation is tightly linked to matrix adhesion, potentially offering unique therapeutic targets. Other studies have also described nonclassical mechanisms of IGF1R signaling, demonstrating that IGF1 ligand binding directly to αvβ3 and α6β4 integrins is necessary for downstream signaling (36, 37) and that FAK, a major signaling kinase localized to matrix adhesions, is activated by IGF1 in HNSCC cells, leading to cell growth and suppressed apoptosis (38, 39).

Part of the signaling architecture in tumor cells is ASK1, a MAP3K that is associated with the receptor complex (17). ASK1 is suppressed via serine and/or threonine and tyrosine phosphorylation when IGF1R kinase is activated (17). Inhibition of the IGF1R by SSTN_IGF1R_ relieves the suppression of ASK-1 and allows its autoactivation and activation of apoptosis via JNK and p38MAPK (17). Downstream signaling from IGF1R also activates cytoplasmic talin, which engages and activates the αvβ3 and αvβ5 integrins involved in cell migration/invasion (19), potentially contributing to their purported roles in apoptosis and IGF1R signaling (36–38).

SSTN_IGF1R_ is remarkably stable *in vivo*, allowing its use in tumor models and potentially as a cancer therapeutic (ref. 17; reviewed in ref. 28). PLA on the PDX models employed here confirms that it acts on its intended target *in vivo*, effectively abolishing the interaction of IGF1R with the integrin coupled to Sdc1, which correlates with activated ASK-1 and reduced growth in the treated tumors. Although not examined here, it is likely that SSTN_IGF1R_ also suppresses the growth of these tumors by reducing angiogenesis. Previous studies have shown that the receptor complex is expressed in endothelial cells during pathologic angiogenesis and that SSTN_IGF1R_ blocks tumor-induced angiogenesis in breast cancer and myeloma models by over 90% (17, 18). Similar to its role in tumor cells, the receptor complex on activated endothelial cells suppresses ASK1 and apoptosis, as described here. It also plays a major role in the activation of VEGFR2 signaling, relying on a mechanism in which the αvβ3 and αvβ5 integrins are coupled via VE-cadherin to activate VEGFR2 (40). The extensive expression of this receptor complex in HNSCC and the positive results obtained here when select HNSCC PDXs were treated with SSTN_IGF1R_*in vivo*, suggest that its signaling mechanisms play a prominent role in this disease.

## Supplementary Material

Supplementary Table S1 Patient characteristics of OPSCC* and ACC** TMAs

## References

[1] Jemal A , Bray F, Center MM, Ferlay J, Ward E, Forman D. Global cancer statistics. CA Cancer J Clin 2011;61:69–90. 21296855 10.3322/caac.20107

[2] Chow LQM . Head and neck cancer. N Engl J Med 2020;382:60–72. 31893516 10.1056/NEJMra1715715

[3] Sharafinski ME , Ferris RL, Ferrone S, Grandis JR. Epidermal growth factor receptor targeted therapy of squamous cell carcinoma of the head and neck. Head Neck 2010;32:1412–21. 20848399 10.1002/hed.21365 PMC2946515

[4] Dale OT , Aleksic T, Shah KA, Han C, Mehanna H, Rapozo DC, . IGF-1R expression is associated with HPV-negative status and adverse survival in head and neck squamous cell cancer. Carcinogenesis 2015;36:648–55. 25896444 10.1093/carcin/bgv053

[5] Lara PC , Bordon E, Rey A, Moreno M, Lloret M, Henriquez-Hernandez LA. IGF-1R expression predicts clinical outcome in patients with locally advanced oral squamous cell carcinoma. Oral Oncol 2011;47:615–9. 21640634 10.1016/j.oraloncology.2011.05.005

[6] Matsumoto F , Fujimaki M, Ohba S, Kojima M, Yokoyama J, Ikeda K. Relationship between insulin-like growth factor-1 receptor and human papillomavirus in patients with oropharyngeal cancer. Head Neck 2015;37:977–81. 24700733 10.1002/hed.23702

[7] Werner H . Tumor suppressors govern insulin-like growth factor signaling pathways: implications in metabolism and cancer. Oncogene 2012;31:2703–14. 21963847 10.1038/onc.2011.447

[8] Parker A , Cheville JC, Lohse C, Cerhan JR, Blute ML. Expression of insulin-like growth factor I receptor and survival in patients with clear cell renal cell carcinoma. J Urol 2003;170:420–4. 12853790 10.1097/01.ju.0000071474.70103.92

[9] Spentzos D , Cannistra SA, Grall F, Levine DA, Pillay K, Libermann TA, . IGF axis gene expression patterns are prognostic of survival in epithelial ovarian cancer. Endocr Relat Cancer 2007;14:781–90. 17914107 10.1677/ERC-06-0073

[10] Matsubara J , Yamada Y, Hirashima Y, Takahari D, Okita NT, Kato K, . Impact of insulin-like growth factor type 1 receptor, epidermal growth factor receptor, and HER2 expressions on outcomes of patients with gastric cancer. Clin Cancer Res 2008;14:3022–9. 18483367 10.1158/1078-0432.CCR-07-1898

[11] Takahari D , Yamada Y, Okita NT, Honda T, Hirashima Y, Matsubara J, . Relationships of insulin-like growth factor-1 receptor and epidermal growth factor receptor expression to clinical outcomes in patients with colorectal cancer. Oncology 2009;76:42–8. 19033715 10.1159/000178164

[12] Valsecchi ME , McDonald M, Brody JR, Hyslop T, Freydin B, Yeo CJ, . Epidermal growth factor receptor and insulinlike growth factor 1 receptor expression predict poor survival in pancreatic ductal adenocarcinoma. Cancer 2012;118:3484–93. 22086503 10.1002/cncr.26661

[13] Donohoe CL , Doyle SL, McGarrigle S, Cathcart MC, Daly E, O'Grady A, . Role of the insulin-like growth factor 1 axis and visceral adiposity in oesophageal adenocarcinoma. Br J Surg 2012;99:387–96. 22241325 10.1002/bjs.8658

[14] Yamamoto T , Oshima T, Yoshihara K, Nishi T, Arai H, Inui K, . Clinical significance of immunohistochemical expression of insulin-like growth factor-1 receptor and matrix metalloproteinase-7 in resected non-small cell lung cancer. Exp Ther Med 2012;3:797–802. 22969971 10.3892/etm.2012.493 PMC3438550

[15] Kurmasheva RT , Houghton PJ. IGF-I mediated survival pathways in normal and malignant cells. Biochim Biophys Acta 2006;1766:1–22. 16844299 10.1016/j.bbcan.2006.05.003

[16] Tao Y , Pinzi V, Bourhis J, Deutsch E. Mechanisms of disease: signaling of the insulin-like growth factor 1 receptor pathway–therapeutic perspectives in cancer. Nat Clin Pract Oncol 2007;4:591–602. 17898809 10.1038/ncponc0934

[17] Beauvais DM , Jung O, Yang Y, Sanderson RD, Rapraeger AC. Syndecan-1 (CD138) suppresses apoptosis in multiple myeloma by activating IGF1 receptor: prevention by SynstatinIGF1R inhibits tumor growth. Cancer Res 2016;76:4981–93. 27364558 10.1158/0008-5472.CAN-16-0232 PMC5010496

[18] Beauvais DM , Ell BJ, McWhorter AR, Rapraeger AC. Syndecan-1 regulates alphavbeta3 and alphavbeta5 integrin activation during angiogenesis and is blocked by synstatin, a novel peptide inhibitor. J Exp Med 2009;206:691–705. 19255147 10.1084/jem.20081278 PMC2699122

[19] Beauvais DM , Rapraeger AC. Syndecan-1 couples the insulin-like growth factor-1 receptor to inside-out integrin activation. J Cell Sci 2010;123:3796–807. 20971705 10.1242/jcs.067645 PMC2964108

[20] Jung O , Trapp-Stamborski V, Purushothaman A, Jin H, Wang H, Sanderson RD, . Heparanase-induced shedding of syndecan-1/CD138 in myeloma and endothelial cells activates VEGFR2 and an invasive phenotype: prevention by novel synstatins. Oncogenesis 2016;5:e202. 26926788 10.1038/oncsis.2016.5 PMC5154350

[21] Rapraeger AC . Synstatin: a selective inhibitor of the syndecan-1-coupled IGF1R-αvβ3 integrin complex in tumorigenesis and angiogenesis. FEBS J 2013;280:2207–15. 23375101 10.1111/febs.12160 PMC3651771

[22] Wang H , Jin H, Beauvais DM, Rapraeger AC. Cytoplasmic domain interactions of syndecan-1 and syndecan-4 with α6β4 integrin mediate human epidermal growth factor receptor (HER1 and HER2)-dependent motility and survival. J Biol Chem 2014;289:30318–32. 25202019 10.1074/jbc.M114.586438 PMC4215216

[23] Wang H , Jin H, Rapraeger AC. Syndecan-1 and syndecan-4 capture epidermal growth factor receptor family members and the α3β1 integrin via binding sites in their ectodomains: novel synstatins prevent kinase capture and inhibit α6β4-integrin-dependent epithelial cell motility. J Biol Chem 2015;290:26103–13. 26350464 10.1074/jbc.M115.679084 PMC4646262

[24] De Rossi G , Evans AR, Kay E, Woodfin A, McKay TR, Nourshargh S, . Shed syndecan-2 inhibits angiogenesis. J Cell Sci 2014;127:4788–99. 25179601 10.1242/jcs.153015 PMC4215719

[25] De Rossi G , Whiteford JR. Novel insight into the biological functions of syndecan ectodomain core proteins. Biofactors 2013;39:374–82. 23559542 10.1002/biof.1104

[26] Whiteford JR , Xian X, Chaussade C, Vanhaesebroeck B, Nourshargh S, Couchman JR. Syndecan-2 is a novel ligand for the protein tyrosine phosphatase receptor CD148. Mol Biol Cell 2011;22:3609–24. 21813734 10.1091/mbc.E11-02-0099 PMC3183016

[27] Whiteford JR , Couchman JR. A conserved NXIP motif is required for cell adhesion properties of the syndecan-4 ectodomain. J Biol Chem 2006;281:32156–63. 16936286 10.1074/jbc.M605553200

[28] Rapraeger AC . Syndecans and their synstatins: targeting an organizer of receptor tyrosine kinase signaling at the cell-matrix interface. Front Oncol 2021;11:775349. 34778093 10.3389/fonc.2021.775349 PMC8578902

[29] Soderberg O , Gullberg M, Jarvius M, Ridderstrale K, Leuchowius KJ, Jarvius J, . Direct observation of individual endogenous protein complexes *in situ* by proximity ligation. Nat Methods 2006;3:995–1000. 17072308 10.1038/nmeth947

[30] Beauvais DM , Burbach BJ, Rapraeger AC. The syndecan-1 ectodomain regulates alphavbeta3 integrin activity in human mammary carcinoma cells. J Cell Biol 2004;167:171–81. 15479743 10.1083/jcb.200404171 PMC2172512

[31] Kimple RJ , Harari PM, Torres AD, Yang RZ, Soriano BJ, Yu M, . Development and characterization of HPV-positive and HPV-negative head and neck squamous cell carcinoma tumorgrafts. Clin Cancer Res 2013;19:855–64. 23251001 10.1158/1078-0432.CCR-12-2746 PMC3581858

[32] Swick AD , Stein AP, McCulloch TM, Hartig GK, Ong IM, Sampene E, . Defining the boundaries and expanding the utility of head and neck cancer patient derived xenografts. Oral Oncol 2017;64:65–72. 28024726 10.1016/j.oraloncology.2016.11.017 PMC5218527

[33] Galvan V , Logvinova A, Sperandio S, Ichijo H, Bredesen DE. Type 1 insulin-like growth factor receptor (IGF-IR) signaling inhibits apoptosis signal-regulating kinase 1 (ASK1). J Biol Chem 2003;278:13325–32. 12556535 10.1074/jbc.M211398200

[34] Hayakawa T , Matsuzawa A, Noguchi T, Takeda K, Ichijo H. The ASK1-MAP kinase pathways in immune and stress responses. Microbes Infect 2006;8:1098–107. 16517200 10.1016/j.micinf.2005.12.001

[35] Yee D . A tale of two receptors: insulin and insulin-like growth factor signaling in cancer. Clin Cancer Res 2015;21:667–9. 25303978 10.1158/1078-0432.CCR-14-2056 PMC4498265

[36] Takada Y , Takada YK, Fujita M. Crosstalk between insulin-like growth factor (IGF) receptor and integrins through direct integrin binding to IGF1. Cytokine Growth Factor Rev 2017;34:67–72. 28190785 10.1016/j.cytogfr.2017.01.003 PMC5401657

[37] Shin DH , Lee HJ, Min HY, Choi SP, Lee MS, Lee JW, . Combating resistance to anti-IGFR antibody by targeting the integrin β3-Src pathway. J Natl Cancer Inst 2013;105:1558–70. 24092920 10.1093/jnci/djt263 PMC3797025

[38] Lehman CE , Spencer A, Hall S, Shaw JJP, Wulfkuhle J, Petricoin EF, . IGF1R and Src inhibition induce synergistic cytotoxicity in HNSCC through inhibition of FAK. Sci Rep 2021;11:10826. 34031486 10.1038/s41598-021-90289-1 PMC8144381

[39] Lehman CE , Khalil AA, Axelrod MJ, Dougherty MI, Schoeff SS, Taniguchi LE, . Antitumor effect of insulin-like growth factor-1 receptor inhibition in head and neck squamous cell carcinoma. Laryngoscope 2020;130:1470–8. 31433065 10.1002/lary.28236

[40] Rapraeger AC , Ell BJ, Roy M, Li X, Morrison OR, Thomas GM, . Vascular endothelial-cadherin stimulates syndecan-1-coupled insulin-like growth factor-1 receptor and cross-talk between αVβ3 integrin and vascular endothelial growth factor receptor 2 at the onset of endothelial cell dissemination during angiogenesis. FEBS J 2013;280:2194–206. 23331867 10.1111/febs.12134 PMC3640762

