# Supplementary material for: Inhibiting IGF1R-mediated Survival Signaling in Head and Neck Cancer with the Peptidomimetic SSTNIGF1R
Source: Cancer Res Commun. 2023 Jan 19;3(1):97–108. doi: 10.1158/2767-9764.CRC-22-0274 (PMC10035507; doi:10.1158/2767-9764.CRC-22-0274)
Supplement: Supplementary Table S1 — Patient characteristics of OPSCC* and ACC** TMAs [file crc-22-0274-s01.docx]

**Supplementary Table SI­­­­­­­­­­­**

**Patient characteristics of OPSCC* and ACC** TMAs**

________________________________________________________

**OPSCC** **ACC**

(n=163) (n=53)

Age at original diagnosis (mean, range) 60 (36-88) 55 (21-82)

Male 129 24

Female 34 29

**Histology**

Squamous Cell Carcinoma 156 0

Adenoid Cystic Carcinoma 0 52

Adenocarcinoma 1 1

Other 4 0

p16+ 86 ND

p16- 44 ND

unknown 31 0

**Stage** (AJCC v7)

T1 54 10

T2 54 7

T3 22 5

T4 13 4

N0 30 31

N1 13 2

N2 103 0

N3 8 0

M0 154 34

M1 0 1

**Tissue Origin**

| Primary | 120 | 42 |
| --- | --- | --- |
| Recurrence | 85 | 17 |
| Lymph node or distant metastasis | 83 | 5 |

___________________________________________________________

Oropharyngeal Squamous Cell Carcinoma*

Adenoid Cystic Carcinoma**

ND = not determined
